# Draft Genomes and Comparative Analysis of Seven Mangrove Rhizosphere-Associated Fungi Isolated From *Kandelia obovata* and *Acanthus ilicifolius*

**DOI:** 10.3389/ffunb.2021.626904

**Published:** 2021-04-14

**Authors:** Chengcheng Shi, Jianwei Chen, Qijin Ge, Jiahui Sun, Wenjie Guo, Jie Wang, Ling Peng, Qiwu Xu, Guangyi Fan, Wenwei Zhang, Xin Liu

**Affiliations:** ^1^BGI-Qingdao, BGI-Shenzhen, Qingdao, China; ^2^BGI Education Center, University of Chinese Academy of Sciences, Shenzhen, China; ^3^Genetic Engineering Research Center, School of Life Sciences, Chongqing University, Chongqing, China; ^4^BGI-Argo Seed Service (Wuhan) Co., Ltd, BGI-Shenzhen, Wuhan, China; ^5^BGI-Shenzhen, Shenzhen, China; ^6^BGI-Fuyang, BGI-Shenzhen, Fuyang, China

**Keywords:** mangrove fungi, genome sequencing, phylogeny, carbohydrate active enzymes, secondary metabolite biosynthesis gene clusters

## Abstract

Mangroves are one of the most productive and biologically diverse ecosystems, with unique plants, animals, and microorganisms adapted to the harsh coastal environments. Although fungi are widely distributed in the mangrove ecosystem and they are playing an important role in the decomposition of organic matter, their genomic profiles are still poorly understood. In this study, we isolated seven Ascomycota fungi (*Westerdykella dispersa* F012, *Trichoderma lixii* F014, *Aspergillus tubingensis* F023, *Penicillium brefeldianum* F032, *Neoroussoella solani* F033, *Talaromyces fuscoviridis* F034, and *Arthrinium marii* F035) from rhizospheres of two mangroves of *Kandelia obovata* and *Acanthus ilicifolius*. We sequenced and assembled the whole genome of these fungi, resulting in size ranging from 29 to 48 Mb, while contig N50 from 112 to 833 Kb. We generated six novel fungi genomes except *A. tubingensis*, and the gene completeness and genome completeness of all seven genomes are higher than 94%. Comparing with non-mangrove fungi, we found Carbohydrate-Binding Modules (CBM32), a subfamily of carbohydrate active enzymes, only detected in two mangrove fungi. Another two subfamilies, Glycoside Hydrolases (GH6) and Polysaccharide Lyases (PL4), were significantly different in gene copy number between *K. obovata* and *A. ilicifolius* rhizospheres (*P*-value 0.041 for GH6, 0.047 for PL4). These findings may indicate an important influence of mangrove environments or hosts on the ability of decomposition in rhizosphere fungi. Secondary metabolite biosynthesis gene clusters were detected and we found the mangrove fungi averagely contain 18 Type I Polyketide (t1pks) synthase, which was significantly higher than 13 in non-mangrove fungi (*P*-value 0.048), suggesting their potential roles in producing bioactive compounds that important for fungi development and ecology. We reported seven mangrove-associated fungal genomes in this study and compared their carbohydrate active enzymes and secondary metabolites (SM) genes with those of non-mangrove fungi, and the results suggest that there are differences in genetic information among fungi in different habitats.

## Introduction

Mangroves forests grow in coastal intertidal zones, with unique plant species populating the saline and brackish water (Ball, 1988). Mangrove trees, together with fishes, crustaceans, and microorganism, make up one of the most productive ecosystems on Earth (Mumby et al., 2004; Serafy et al., 2015; Hamilton and Friess, 2018). The mangrove ecosystem is distributed along tropical coasts in more than 100 countries (Giri et al., 2011) and plays key role in supporting human society with food, shelters, and livelihoods (Hochard et al., 2019). With the importance of mangrove ecosystems (Alongi, 2014), it would be critical to explore the genomic mechanisms underlying their adaptation and high production.

Fungi, as heterotrophs, play role as decomposers to degrade the organic matters in ecosystem. In addition, fungi, especially the rhizosphere fungi, can interact with plants to form mycorrhizal symbionts, promoting the nutrient metabolisms in plants thus affecting the plant growth in the different environments (Perotto and Bonfante, 1997; van der Heijden, 2016). It has been previously reported that different conditions of soil, including tillage shifts, depths, plant species, and environmental stresses, have obvious impacts on fungal diversity and their biological functions (Berg et al., 2005; Costa et al., 2006; Wang et al., 2017; Giard-Laliberté et al., 2019; Vanegas et al., 2019; Zhang et al., 2019). At the same time, fungi produce secondary metabolites (SM) including bioactive compounds to respond to the environments and secure their ecological niches (Keller, 2019). Thus, fungi represent an important source for the SM researches and natural bioactive compounds development. Considering the highly productive and diversified ecosystem of mangroves, it would be important to investigate the function of fungi that live in these transitional intertidal ecosystem and secondary metabolism for further applications.

To date, over 200 fungal species have been found associated with mangrove plant roots (Thatoi et al., 2013a,b; Alsheikh-Hussain et al., 2014). Recent studies on mangrove associated fungi mainly applied molecular markers or metagenomic methods to reveal the differences in fungal composition and diversity under different rhizosphere conditions of mangroves (Simões et al., 2015; Sanka Loganathachetti et al., 2017; Shyamalina Haldar, 2019; Vanegas et al., 2019). For example, the metagenomic datasets from mangrove rhizosphere revealed that Ascomycota was the dominant phylum and Basidiomycota was less abundant fungi (Simões et al., 2015, Shyamalina Haldar, 2019). Also, a research on fungal communities of different soil compartments in mangrove ecosystem reported that the rhizosphere had significantly lower fungal species richness than bulk soil samples and different soil compartments significantly affected the fungal community composition (Sanka Loganathachetti et al., 2017). These studies on mangrove fungi revealed their characteristics, but there are still more fungal species to be explored for taxonomic locations and functional capacities. The genomic mechanisms of adaptation to the complex costal environments, as well as the symbiosis between plants and fungi, remain largely unexplored because of limited genomic resources of mangrove fungi.

In this study, we isolated three fungal species (F012, F014, and F034) from *Kandelia obovata* rhizosphere and four fungal species (F023, F032, F033, and F035) from *Acanthus ilicifolius* rhizosphere. We then carried out whole genome sequencing (WGS) and genome assembly of these fungi to find species including *Westerdykella dispersa, Trichoderma lixii, Talaromyces fuscoviridis, Penicillium brefeldianum, Aspergillus tubingensis, Neoroussoella solani*, and *Arthrinium marii*, all of which belong to the phylum Ascomycota. We compared the whole genomes to depict possible roles of functional carbohydrate active enzymes and SM related gene clusters in environmental adaptations.

## Materials and Methods

### Sampling and Isolation

The mangrove rhizosphere soil samples from one *K. obovata* tree and one *A. ilicifolius* tree were collected by the 1–2 mm soil tightly adhered to the 10–20 cm underground roots from East Harbour National Nature Reserve (Hainan, China) in April 13, 2018. The 5 g rhizosphere soil of each sample was put into a conical flask containing 100 ml sterile water and cultured by shock for 1 h. The soil suspension was diluted 100 times, and then 0.2 ml of the mixed suspension was added to the Potato Dextrose Agar (PDA media, purchased from HOPEBIO, China). The single colonies were toke from coated plate and then purified the single colony again. After purified, the isolates were inoculated in PDA media and incubated in a 28°C constant temperature incubator for 2–5 day until the mycelium growth was observed and the colony characteristics were documented. In this way, three fungal species were isolated from the rhizosphere soil of *Kandelia obovata*, and four fungal species were isolated from the rhizosphere soil of *A. ilicifolius*. The strains were then deposited at China National GeneBank (Qingdao), BGI-Qingdao, China.

### DNA Extraction and Sequencing

The total DNA of each fungal strain was extracted using the CTAB (cetyl trimethylammonium bromide) method (Healey et al., 2014). MGIEasy Micro DNA Library Preparation Kit (TM) (MGI, cat. No. 1000011553) was used for DNA fragmentation and PCR amplification through *Tn5* transposase for each sample following the manufactures instructions. The amplification products were then purified and quantified, and subjected to a single-strand circular DNA library preparation. All libraries for WGS were sequenced on the BGISEQ-500 platform (BGI-Qingdao, China) to produce 100 bp pair-end raw reads.

### Genome Assembly and Species Identification

The raw reads of low quality and resulted from PCR duplication were filtered using SOAPnuke (v1.6.5) (Chen et al., 2018) with parameters of “-q 0.2 -l 15 -n 0.05 -d.” The obtained clean reads were assembled into contigs and scaffolds using SPAdes (v3.10.1) (Bankevich et al., 2012) with a range of kmer lengths from 33 to 83 by step size of 10. For the assembled genomes, we used BUSCO (v3.0.2) (Waterhouse et al., 2018) in genome mode with the closest evolutionary database to evaluated the completeness.

We predicted the rRNAs in the assembled genomes using RNAmmer (v1.2) (Lagesen et al., 2007). Aligning the annotated internal transcribed spacer (ITS) sequences of the assembled genomes to a type-derived ITS database [PRJNA177353: Fungal Internal Transcribed Spacer RNA (ITS) RefSeq Targeted Loci Project] by blastn (v2.6.0), we assigned the species of ITS sequence with best identity (>98.5%) to our assembled species. Thus, the fungal strains F012, F014, F034, F023, F032, F033, and F035 were identified as *Westerdykella dispersa, Trichoderma lixii, Talaromyces fuscoviridis, Aspergillus tubingensis, Penicillium brefeldianum, Neoroussoella solani*, and *Arthrinium marii*, respectively (Supplementary Table 1).

### Genome Annotation and Function Classification

We used RepeatMasker-open-4-0-6 (A.F.A. Smit^1^; Friess et al., 2019) to annotate the repeats in the assembled genomes (using parameters of “-nolow -no_is -norna”). First, we used RepeatModeler-open-1-0-8 (A.F.A. Smit^2^; Bryan-Brown et al., 2020) with default settings to predict the repeats and together with the Repbase library, a homolog search of repeats was then carried out to predict the final repeats in the genomes. Following, we predicted protein coding genes using a pipeline combining *ab initio* and homology-based predictions. The *ab initio* prediction was performed using Augustus (v3.2.1) (Stanke et al., 2006) and GeneMark (v4.32) (Lomsadze et al., 2005) with parameters of “-ES -fungus -cores 10.” Homologous proteins were downloaded from NCBI (Supplementary Table 2), and aligned to the genomes using BLAT (v.36) (Kent, 2002). Alignments with over 70% coverage were retained and the respective aligned regions were extracted for further gene structure prediction by GeneWise (v2.4.1) (Birney et al., 2004). The *ab initio* predictions and the homolog based gene predictions were merged to obtain the final non-redundant consensus gene set using EvidenceModeler pipeline (Haas et al., 2008).

Subsequently, functional annotation of the predicted genes was carried out by aligning the predicted proteins to KEGG (Kanehisa and Goto, 2000), KOG Clusters of orthologous groups for eukaryotic complete genomes (Tatusov et al., 2003), TrEMBL, and Swiss-Prot (UniProt, 2015) using BLASTP with *E*-value of 1e-5. Protein domains were searched using InterProScan (v5.3) (Jones et al., 2014). We evaluated the completeness of the predicted gene sets using BUSCO (v3.0.1).

Meanwhile, the protein sequences were aligned against the CAZy (v20190731) (Cantarel et al., 2009) database using dbCAN2 (v20190908) (Zhang et al., 2018) by HMMER (v3.1b2) (Eddy, 2009) with *E*-value < 1e-15 to detect carbohydrate active enzymes. The secondary metabolite biosynthesis gene clusters of all the assemblies were detected using antiSMASH (v4.0) (Medema et al., 2011) with default parameters. Wilcox-Test in R software (v3.1.1) was used to analyze significant differences of carbohydrate active enzymes and secondary metabolite biosynthesis gene clusters between mangrove fungi (seven sequenced here and one reported HXQ-H-1, Peng et al., 2019) and non-mangrove fungi (Supplementary Table 2), as well as between fungi from two mangrove rhizospheres (*K. obovata* and *A. ilicifolius*) in this study.

### Phylogenetic Analysis

The whole genome sequences and protein sequences of 23 related fungi species were analyzed together with the seven fungi we have assembled (Supplementary Table 2). Core genes of all the 30 genomes were predicted using CD-Hit (v4.6.6) (Fu et al., 2012) with parameters of “-c 0.4 -n 2 -p 1 -g 1 -d 0 -s 0.5 -aL 0.5 -aS 0.5.” The core genes which presented in all genomes were selected for multiple sequence alignment by MUSCLE (v3.8.1). Then a phylogenetic tree was obtained based on the alignment using TreeBeST (v19.2) (Guindon et al., 2003) with parameters of “phyml -b 100.” Gene families were identified using a workflow of all-vs.-all BLASP followed by clustering by OrthoMCL (Li et al., 2003) with default parameters. Single-copy orthologous genes of these 30 species were extracted to construct maximum likelihood phylogenetic tree using IQ-TREE (Nguyen et al., 2015) with GTR model (a commonly used substitution model in construction of phylogenetic trees).

## Results

### Genome Sequencing and Assembly

The obtained data of seven fungi sequenced in this study ranges from 4.03 to 7.89 Gb, with about 6.24 Gb (162-fold coverage) in average. With these data, we assembled draft genomes using SPAdes software (see section Materials and Methods, Table 1). The assembled sizes of the seven fungal genomes ranged from 29.39 Mb (*W. dispersa*) to 48.27 Mb (*N. solani*), comparable to those of the closely related species, as shown in Supplementary Table 2. The scaffold N50 of the seven fungal genomes ranged from 112.3 Kb (*T. fuscoviridis*) to 941.4 Kb (*T. lixii*), reflecting relatively good completeness and continuity of these genome sequences. The contig N50 of these assemblies were longer than 300 Kb except *T. fuscoviridis* (contig N50 112.3 Kb), which can meet requirements of gene annotation and downstream analysis. The GC contents of these draft genomes ranged from 47.1% (*T. fuscoviridis*) to 54.3% (*A. marii*).

**Table 1:** The sequences and assembly statistics of the seven fungi.

**Fungi**	**Clean data (Gb)**	**Assembled size (Mb)**	**Scaffold number**	**Scaffold N50 (Kb)**	**Contig N50 (Kb)**	**GC content (%)**	**Mapping ratio (%)**
*Westerdykella dispersa* F012	4.03	29.39	124	912.80	727.84	52.7	96.8
*Trichoderma lixii* F014	5.20	40.83	448	941.43	832.94	49.1	97.0
*Aspergillus tubingensis* F023	7.89	37.19	870	814.52	717.45	49.4	98.1
*Penicillium brefeldianum* F032	5.77	32.55	300	328.24	324.48	51.5	93.4
*Neoroussoella solani* F033	7.09	48.27	360	429.66	429.66	48.8	97.3
*Talaromyces fuscoviridis* F034	6.88	36.19	2,548	112.30	112.30	47.1	97.8
*Arthrinium marii* F035	6.79	45.86	139	612.44	612.44	54.3	98.5

**Figure 1:**
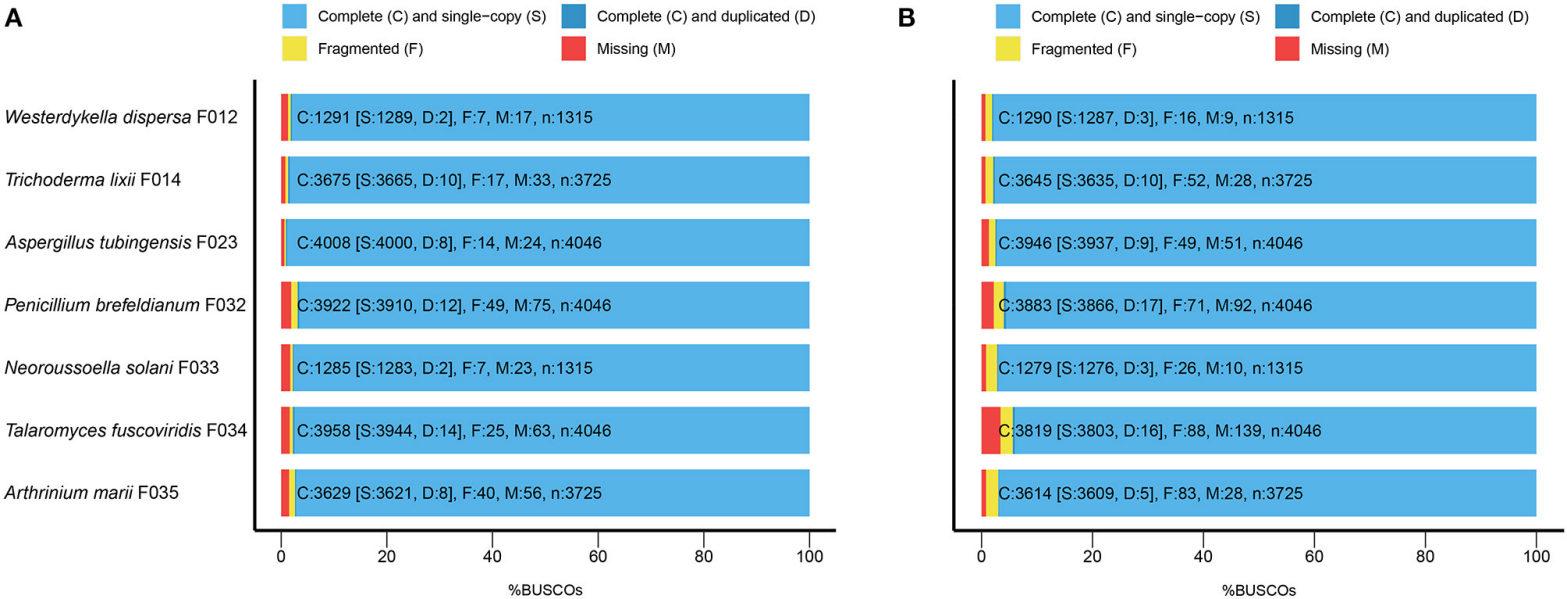
The BUSCO assessment of the seven fungal genome assemblies. For *Trichoderma lixii* F014, *Neoroussoella solani* F033, *Talaromyces fuscoviridis* F034, *Aspergillus tubingensis* F023, *Penicillium brefeldianum* F032, *Westerdykella dispersa* F012, *Arthrinium marii* F035, we present **(A)** the BUSCO scores of the genome assemblies and **(B)** the BUSCO scores of the predicted gene sets.

To evaluate the completeness of the assemblies, we mapped the sequenced data back to the genomes and found that their mapping ratio were higher than 96% (Table 1). To further assess the completeness in gene regions, we carried out BUSCO analysis and found that 97% of the conserved fungi genes could be covered in our genome assemblies (Figure 1A, Supplementary Table 2), indicating high completeness and good quality of the draft genomes.

### Genome Annotation

**Figure 2:**
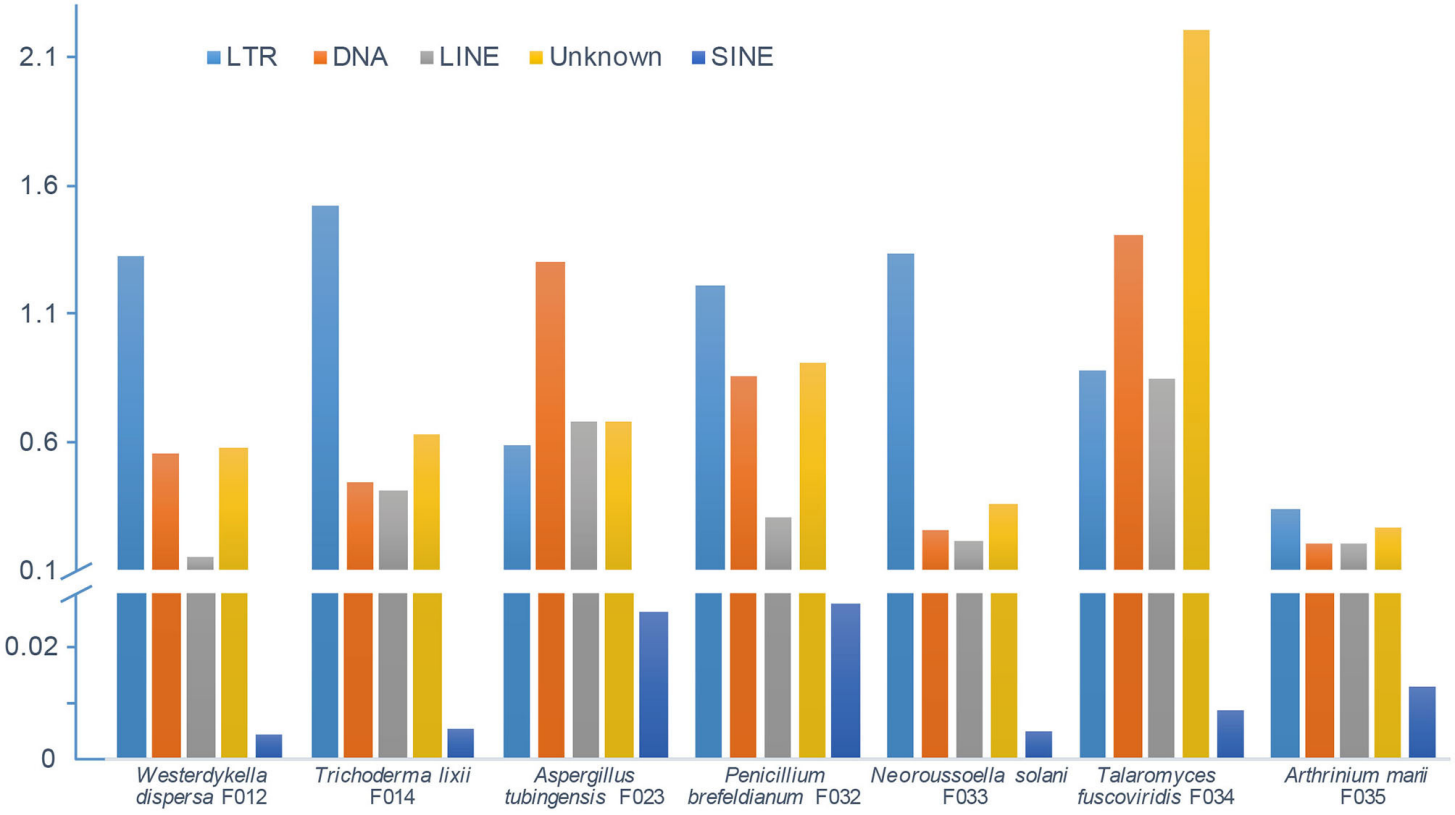
Repeat contents of seven fungal genomes. Bars indicate different repeats, including LTR (long terminal repeat element, light blue), DNA (DNA transposon, orange), LINE (long interspersed nuclear element, gray), unknown repeats (dark yellow), and SINE (short interspersed nuclear element, blue).

In order to depict and compare different contents of the assembled fungal genomes, we first predicted the repeat elements. These fungal genomes had low repeat contents, ranging from 1 to 5% (Figure 2). In detail, we predicted 0.68 Mb (2.30%), 1.17 Mb (2.88%), 1.16 Mb (3.11%), 1.01 Mb (3.11%), 1.03 Mb (2.13%), 1.90 Mb (5.26%), and 0.46 Mb (1%) repeats in genomes of *W. dispersa, T. lixii, A. tubingensis, P. brefeldianum, N. solani, T. fuscoviridis*, and *A. marii*, respectively. Interestingly, we found that *T. fuscoviridis* has the highest proportion of repeat sequences (5.26%), of which more than one-third were unknown repeats (~2.2%). While in *W. dispersa, T. lixii, P. brefeldianum, N. solani*, and *A. marii*, long terminal repeats (LTRs) was the most abundant, followed by the DNA transposons and long interspersed nuclear elements (LINEs). Three major categories of repeats including LTRs, DNA transposons, and LINEs made up more than 75% of all repeats in six fungi genomes (*W. dispersa, T. lixii, P. brefeldianum, A. tubingensis, N. solani*, and *A. marii*), while short interspersed nuclear elements (SINEs) made up the smallest proportion (Figure 2).

Following the annotation of the repeats, we predicted protein coding genes and annotated functions (Table 2). In average, there are around 10,000 genes in *W. dispersa* (9,922), *T. lixii* (12,260), *A. tubingensis* (11,818), *P. brefeldianum* (10,448), *N. solani* (15,859), *T. fuscoviridis* (12,804), and *A. marii* (14,346) genomes. We found that the gene models of these seven fungi are similar, with respect to transcript length (~1.7 Kb), coding sequence length (~1.5 Kb), exon length (~504 bp), and intron length (~103 bp). To assess the completeness of the predicted gene sets, we performed BUSCO to evaluate them, finding that at least 94% of the conserved orthologous genes were predicted (Table 2 and Figure 1B). In addition, we mapped the predicted protein sequences against six databases (NR, KEGG, KOG, TrEMBL, Swiss-Prot, and InterPro) to find that more than 90% of the proteins in all fungal genomes had well-aligned homologs with known functions. Thus, we were able to assign putative functions to these genes. Overall, the BUSCO evaluation and functional annotation results suggest that the gene sets can be used for downstream analysis.

**Table 2:** The protein coding gene models predicted in the seven fungal genomes sequenced.

**Type**	***Westerdykella dispersa* F012**	***Trichoderma lixii* F014**	***Aspergillus tubingensis* F023**	***Penicillium brefeldianum* F032**	***Neoroussoella solani* F033**	***Talaromyces fuscoviridis* F034**	***Arthrinium marii* F035**
Gene number	9,922	12,260	11,818	10,448	15,859	12,804	14,346
Average gene length (bp)	1717.31	1713.82	1678.89	1756.38	1642.93	1765.98	1687.53
Average coding length (bp)	1530.82	1515.27	1490.82	1517.38	1473.19	1515.13	1480.34
Average exon number	2.94	2.85	3.12	3.13	2.94	3.16	2.79
Average exon length (bp)	521.52	532.27	478.41	485.04	500.24	478.82	530.01
Average intron length (bp)	96.37	107.51	88.87	112.29	87.27	115.90	115.55
BUSCO completeness (%)	98.1	97.9	97.5	95.9	97.2	94.3	97.0
Functionally annotated (%)	93.18	98.00	98.98	97.89	92.46	96.77	90.61
Mapped to NR (%)	92.59	97.54	98.97	97.82	91.92	96.56	89.85
Mapped to Swiss-Prot (%)	64.12	65.16	68.06	69.53	60.70	65.32	57.88
Mapped to KEGG (%)	67.17	69.55	71.49	72.97	65.88	69.31	63.06
Mapped to KOG (%)	52.11	52.37	54.30	55.71	47.72	52.34	45.94
Mapped to TrEMBL (%)	92.56	97.95	98.95	97.81	91.97	96.38	89.81
Mapped to InterPro (%)	78.18	80.41	82.92	84.02	77.46	80.78	74.95

### Phylogeny Analyses

**Figure 3:**
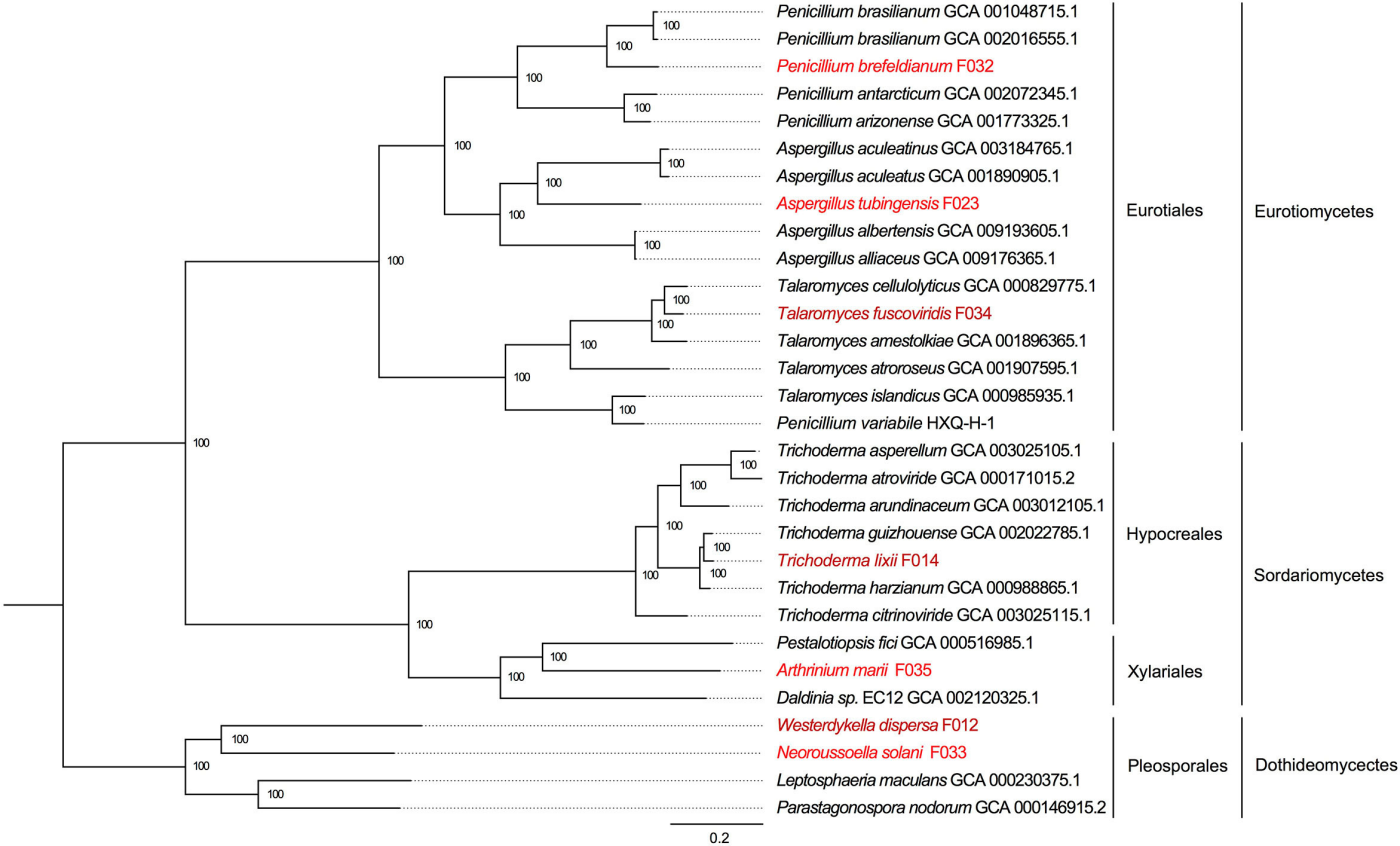
The phylogenetic tree based on single copy genes and location of the seven mangrove fungi. The three classes and four orders are marked on the right, and the mangrove fungi isolated from *Kandelia obovata* and *Acanthus ilicifolius* are indicated in dark red and red.

To reveal the phylogenetic positions of the seven isolated fungi, we further compared them with 23 public fungal genomes to obtain the phylogeny with two strategies (see section Materials and Methods). We identified 610 core genes shared among all 30 genomes. At the same time, we also carried out gene family clustering of them and identified 2,059 gene families with single copy in each genome (single-copy gene families). Using the core genes and single-copy gene families, we constructed two phylogenetic trees, respectively. We found the two phylogenetic trees were extremely consistent, with all the nodes to be fully supported by bootstrapping (bootstrap value ~100%, as shown in Figure 3 and Supplementary Figure 1). In the phylogeny trees, three clades represented the classes of Eurotiomycetes, Sordariomycetes, and Dothideomycetes, respectively. In the class of Eurotiomycetes, *A. tubingensis, P. brefeldianum*, and *T. fuscoviridis* were close to the fungi from the same order of Eurotiales including families of Aspergillaceae and Trichocomaceae. The class of Sordariomycetes here had two orders, in which *A. marii* and *Pestalotiopsis fici* were most closely related species belonging to Xylariales, while *T. lixii* and *Trichoderma guizhouense* were sister species belonging to Hypocreaceae. In the class of Dothideomycetes, *W. dispersa* and *N. solani* were in sister families, both of which neighbored to Leptosphaeriaceae and Phaeosphaeriaceae belonging to Pleosporales. We found the phylogeny indicated here was consistent with the expectations of the ITS based species identification.

### Carbohydrate Active Enzymes

**Figure 4:**
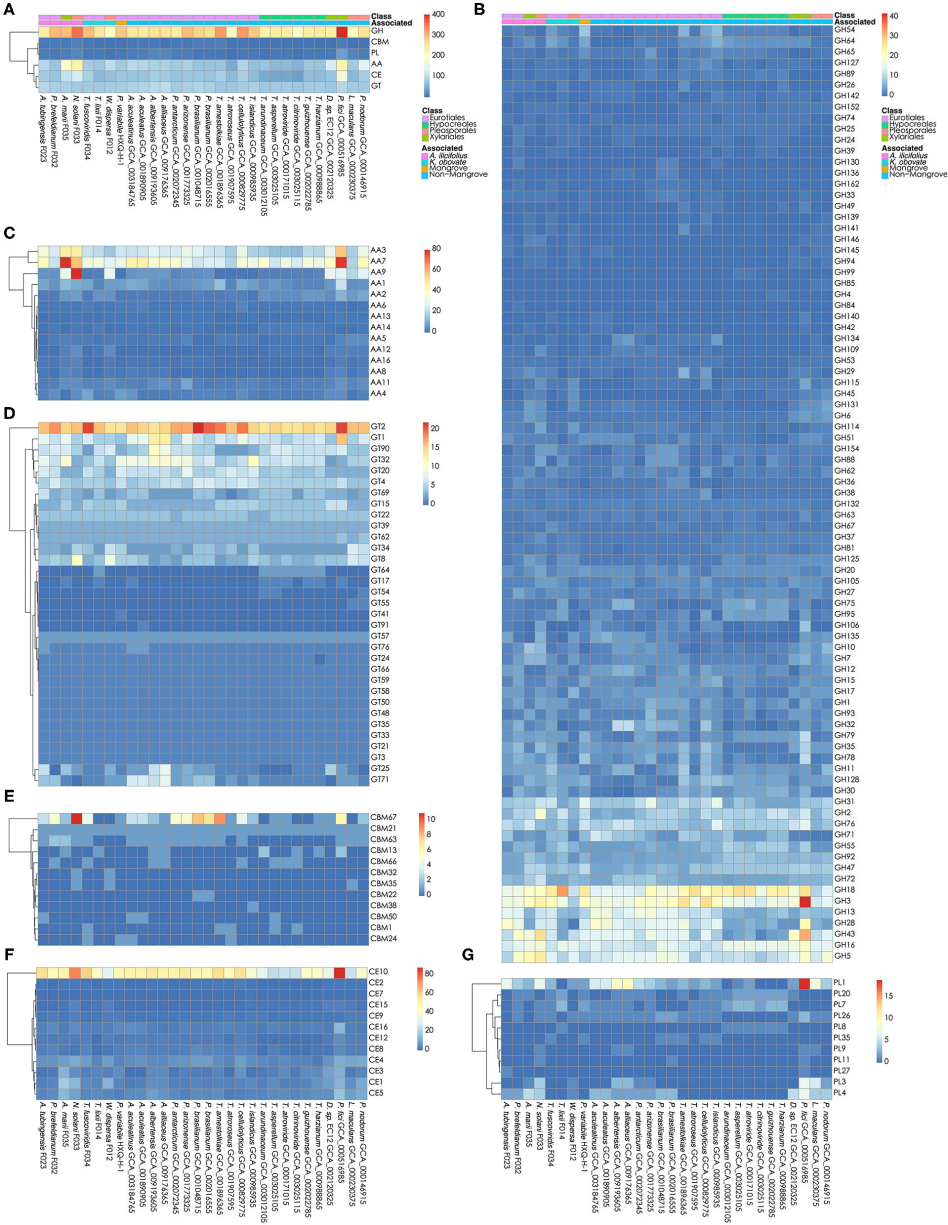
Carbohydrate active genes in the 30 fungal genomes. The number of CA genes in all six classes **(A)**; glycoside hydrolases, GHs **(B)**; auxiliary activities, AAs **(C)**; glycosyl transferases, GTs **(D)**; carbohydrate-binding modules, CBMs **(E)**; carbohydrate esterases, CEs **(F)**; and Polysaccharide Lyases, PLs **(G)** are shown, respectively.

The carbohydrates metabolism is essential for fungi. We analyzed the carbohydrate active enzymes (CAZys) in the *W. dispersa* (475), *T. lixii* (489), *A. tubingensis* (543), *P. brefeldianum* (564), *N. solani* (832), *T. fuscoviridis* (576), and *A. marii* (712), respectively (Supplementary Table 3, Figure 4, and Supplementary Figure 2). In detail, there were six classes of enzymes and modules including Auxiliary Activities (AAs, 14 subfamilies), Carbohydrate Esterases (CEs, 12 subfamilies), Glycosyl Transferases (GTs, 33 subfamilies), Glycoside Hydrolases (GHs, 85 subfamilies), Polysaccharide Lyases (PLs, 11 subfamilies), and Carbohydrate-Binding Modules (CBMs, 12 subfamilies) (Supplementary Table 4). We found similar distributions of gene numbers of these six classes in all the seven fungi, with four classes of GH (about 278 genes on average), AA (about 125 genes on average), CE (about 89 on average), and GT (about 88 on average) to be most abundant.

Interestingly, we found significant differences of gene numbers in some CAZys subfamilies when comparing mangrove with non-mangrove fungi, as well as comparing mangrove fungi from two rhizospheres. Comparing the mangrove fungi with non-mangrove ones, we identified significantly different two subfamilies, CBM32 (*P*-value, 0.020) and GH146 (*P*-value, 0.028), with more copy number in mangrove fungi. The CBM32 genes were only found in *N. solani* and *W. dispersa*. GH146 was found more than one gene in *A. tubingensis, A. marii, N. solani*, and *W. dispersa*, while only one copy in three non-mangrove fungi (*Leptosphaeria maculans* GCA_000230375, *Parastagonospora nodorum* GCA_000146915, and *Pestalotiopsis fici* GCA_000516985). Moreover, subclasses of GH6 (*P*-value, 0.042) and PL4 (*P*-value, 0.048) were significantly different between groups of fungi isolated from the two mangrove rhizospheres. As for GH6, we found only one gene in each of the *K. obovata* related fungi, comparing to at least two in the *A. ilicifolius* related. Similarly, for PL4, we found it even missing in two *K. obovata* related fungi (Supplementary Table 4).

### Secondary Metabolite Biosynthesis Gene Clusters

**Figure 5:**
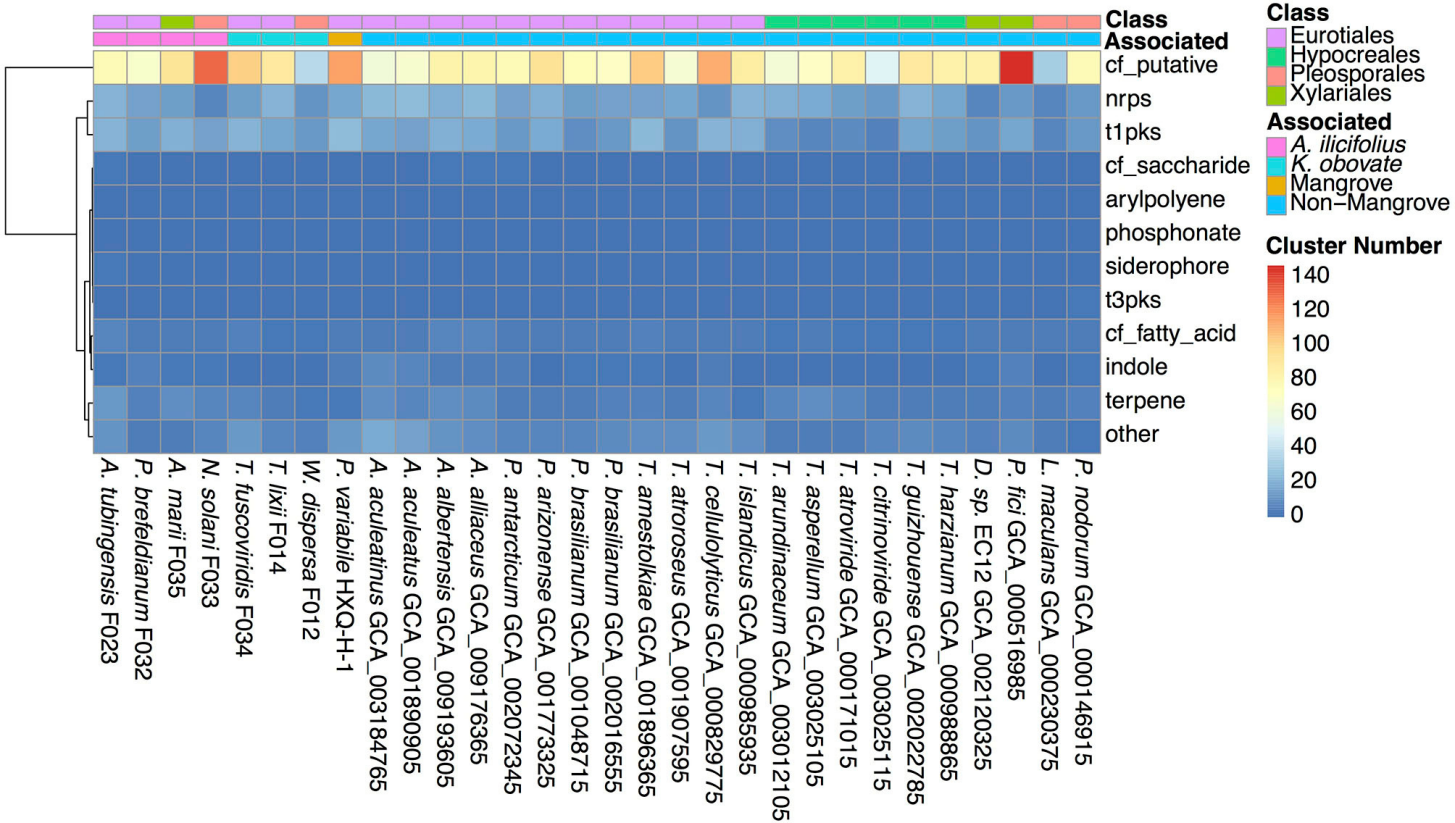
The secondary metabolite biosynthesis gene clusters in the 30 fungal genomes. The information of classes and groups are shown on the top by different colors.

Secondary metabolites are important for fungal development, environmental response, and ecological niches (Keller, 2019), thus we analyzed the SM biosynthesis gene clusters in the investigated fungi. By searching through the antiSMASH database, SM biosynthesis gene clusters in *W. dispersa* (72), *T. lixii* (143), *T. fuscoviridis* (163), *A. tubingensis* (157), *P. brefeldianum* (115), *N. solani* (174), and *A. marii* (149) were identified, respectively (Figure 5, Supplementary Table 5). These gene clusters were further classified into 12 categories according to the types of the SM, including Aryl polyene cluster (arylpolyene), Possible fatty acid cluster (cf_fatty_acid), Putative cluster of unknown type (cf_putative), Possible saccharide cluster (cf_saccharide), Indole cluster (indole), Non-ribosomal peptide synthetase cluster (nrps), Phosphonate cluster (phosphonate), Siderophore cluster (siderophore), Type I Polyketide synthase (t1pks), Type III Polyketide synthase (t3pks), Terpene (terpene), and other (a cluster that contained a secondary metabolite related protein that does not fit into any other category) (Figure 5, Supplementary Table 5). Among the different classes of SM gene clusters in the seven fungi, gene clusters of cf_putative, nrps, t1pks, terpene, and others were the most represents (Figure 5). Moreover, we observed differences in SM gene clusters between mangrove and non-mangrove fungi (Supplementary Figure 3), as well as between the *K. obovata* and *A. ilicifolius* rhizosphere fungi (Supplementary Figure 4). Genes coding t1pks were more common in the mangrove fungi than non-mangrove, with an average of 18 and 13 t1pks, respectively, showing significant difference (*P*-value, 0.048). Comparing the fungi derived from *K. obovata* and *A. ilicifolius* rhizospheres, we found the indole gene clusters to be significantly different (*P*-value, 0.042). In the three fungi from *K. obovata* rhizosphere, the indole was absent in two genomes, and there was only one cluster in the remaining fungi. While in four *A. ilicifolius* rhizosphere fungi, we identified at least two indole clusters in each genome.

## Discussion

### Diversity of Fungal Species in Mangrove Rhizosphere

Diversity of rhizosphere fungal communities is associated with soil, host plants, and environmental factors (Becklin et al., 2012; Bonito et al., 2014; Krüger et al., 2017). Meanwhile, fungal compositions and diversities have been previously proved to be different in mangrove rhizosphere samples (Yu et al., 2015). Additionally, according to high-throughput amplicon sequencing studies, more than 100 fungi species could be detected from different mangroves rhizospheres (Yao et al., 2019), but many fungi cannot successfully grow in laboratory culture condition (Glynou et al., 2018), which often introduce the species bias or loss during the isolation. We isolated seven fungi belonging to different Ascomycota genera in mangrove rhizosphere samples, and there might be more than seven fungal species in mangrove rhizosphere.

### CAZys Related to Degradation of Plant Cell Walls in Mangrove Fungi

Glycoside Hydrolases (GHs) and Polysaccharide Lyases (PLs) are both active enzymes to catalyze the cleavage of glycosidic bonds, which is essential for polysaccharide degradation. The GHs (EC 3.2.1.-) function through hydrolysis or rearrangements, and they are the major members of cellulase (e.g., EC 3.2.1.4, EC 3.2.1.91, EC 3.2.1.21) (Sukharnikov et al., 2011), hemicellulose (e.g., EC 3.2.1.8, EC 3.2.1.37, EC 3.2.1.131, EC 3.2.1.55), and pectinase (e.g., EC 3.2.1.15). These enzymes are important for hydrolyzing cell walls of plants. Due to the sequence characteristics of simple domain organization of fungal active enzymes, their ability to hydrolyze polysaccharides is different from the one described in bacteria, and often affected by their morphological characteristics and environment (Berlemont, 2017). The CAZy results suggested that GHs accounted for the most among the six classes of CAs (42–54%, Supplementary Table 3), with more than 80 GH subfamilies detected in the seven mangrove fungi (Supplementary Table 4). In particular, there were two subfamilies GH146 and GH6 that have significant difference in copy numbers between mangrove and non-mangrove fungi, and between *A. ilicifolius* and *K. obovata* associated fungi, respectively. Especially GH6 is a family of cellobiohydrolase (EC 3.2.1.91). Although the four fungi of *A. ilicifolius* (F023, F032, F033, and F035) rhizosphere come from three different orders, their average gene number of GH6 is twice than that of *K. obovata* rhizosphere fungi. This might reflect convergent evolution or composition in fungal community of different species in similar environments.

The PLs (EC 4.2.2.-) are a class of enzymes that catalyze the cleavage of uronic acid-containing polysaccharide chains without hydrolysis. The PL class contains 40 subfamilies, of which only 11 (PL1, PL11, PL20, PL26, PL27, PL3, PL35, PL4, PL7, PL8, and PL9) were detected in the seven mangrove fungi genomes. PL1 and PL3 genes are important pectinase (pectin and pectate lyases, EC 4.2.2.10, EC 4.2.2.2) that catalyze break-down of pectin, another primary component of plant cell walls. The gene number of PL1 subfamily in *A. tubingensis* F023 was comparable to those in other *Aspergillus* fungi, and this subfamily has more genes than other PL subfamilies. Therefore, it may be a dominant fungal genus involved in pectin metabolism in the mangrove environment. The PL4 is a class of rhamnogalacturonan endolyase (EC 4.2.2.23). Previous study reported that PL4 has a different structure with pectate lyases, and its action requires pH environment and assistance of rhamnogalacturonan acetyl-esterases (van den Brink and de Vries, 2011). The difference of PL4 in two mangrove rhizospheres, and significant difference of GHs subfamilies associating cellulase in mangrove fungi, indicate the influence of diverse mangrove environments and hosts on both the fungi community and their genomic feature of CAs, probably resulting in variable ability of decomposing plant cell walls in the mangrove habits.

Unlike enzymes, CBMs are characterized by a domain that binds to carbohydrate in carbohydrate active enzymes. There are 86 known CBM families, and CBMs are often found in carbohydrate active enzymes such as GHs and PLs (Consortium, 2017). In this study, we found 12 CBM families in these 30 fungi genomes, of which CBM32 was only detected in two mangrove fungi. CBM32 genes were previously found in bacteria and eukaryotes with the function to bind to various substrates, showing high complex diversity in different carbohydrate catalytic enzymes (Abbott et al., 2007). The uniqueness of CBM32 in mangrove fungi found here indicated their possibly important function for mangrove habitat metabolism, waiting for further experimental validations, and detailed studies.

### Biological and Application Prospect of SM in Mangrove Fungi

Previous studies on rhizosphere fungi from *Trichoderma, Talaromyces*, or *Aspergillus* revealed important roles of SM in antibiotics, plant–microbe interactions, and regulations (Contreras-Cornejo et al., 2016; Zhai et al., 2016; Orfali and Perveen, 2019). For example, the fungi *Trichoderma* are representative ascomycetes fungi with strong adaptability living in soil environment. Compound ethylene in *Trichoderma atroviride* can regulate cell differentiation and defense responses in plant (Contreras-Cornejo et al., 2015a). Isoprenoid abscisic acid (ABA) from *T. atroviride* and *Trichoderma virens* have effect on regulating stomatal aperture in *Arabidopsis thaliana* (Contreras-Cornejo et al., 2015b). Indolic compounds could control plant growth and development processes, such as root growth and inducing formation of adventitious root in *A. thaliana* (Contreras-Cornejo et al., 2009). However, compositions and expression of SM biosynthesis genes were found to be highly associated with different environments (Aleti et al., 2017; Keller, 2019). In this study, we observed different indole clusters in two mangrove rhizospheres. This may be related to the root growth of different mangrove species. Living in the mangrove environments, the interaction between rhizosphere fungi and mangrove plants which have strong root system may be more complicated. With the whole genomes of the isolated fungi, the depicted bioactive compounds diversity in SM biosynthesis gene clusters between different environments would help us to understand the expression, regulation, and interaction of the SM in the mangrove ecosystem.

Secondary metabolites offer great potential for biomedicine drug development (Thatoi et al., 2013a; Ancheeva et al., 2018; Deshmukh et al., 2018; Nicoletti et al., 2018). Many extracts from mangrove fungi have been reported with antiviral, antibacterial, or anticancer properties. For instance, a natural product extracted from the mangrove rhizosphere fungus *Aspergillus terreus* has a notable antiviral activity against H1N1 (Gao et al., 2013). Other compounds extracted from the mangrove rhizosphere soil fungi *Penicillium chrysogenum* and *Sarocladium kiliense* showed cytotoxicity against the HeLa cell lines (Guo et al., 2015; Li et al., 2018). These results suggest that fungi living in the mangrove ecosystem represent a precious source of novel bioactive compounds. Among the SM biosynthetic genes, pks, and nrps are most studied ones. They are dramatically associated with the expression of biomedicine related SMs (Koehn and Carter, 2005). The pks catalyzes the synthesis of peptides, which is one of the largest classes of SM including macrolides, tetracyclines, anthraquinones, polyethers, and so on. They generally have antibacterial, antifungal, antitumor, and immunosuppressive function. In these mangrove fungi, we also reported that type I pks genes (t1pks) are particularly abundant. This might reflect the characteristics of mangrove fungal species, including the expression of genes that might be upregulated to adapt themselves to a harsh environment, which could be exploited for the development of new drugs. Even though most of the secondary metabolic genes are silent in general experiment condition (Ancheeva et al., 2018), the identified SM biosynthesis gene clusters based on whole genomes especially in the mangrove fungi could be the importantly basic resource for further development (Brakhage and Schroeckh, 2011).

## Conclusion

Mangroves represent one of the most productive ecosystems on Earth. Unfortunately, these mangrove ecosystems are currently under serious threats due to climate changes and human activities. Besides protecting the shrinking mangrove ecosystems, it is also important to study and understand the biodiversity of the mangrove ecosystems. In this study, we successfully isolated and sequenced seven fungal species from mangrove *K. obovata* and *A. ilicifolius* rhizosphere samples. Using WGS, we assembled draft genomes of these seven species. They are differently distributed in the phylogeny, from three classes, four orders, six families, and seven genera. We identified the carbohydrate active enzymes and secondary metabolite biosynthesis gene clusters for the seven fungi. Comparing with non-mangrove fungi, the CAZys subfamilies of CBM32 only detected in two mangrove fungi. The subfamily PL4 related to cell wall hydrolysis has more than two copies in mangrove fungi from *A. ilicifolius*, higher than fungi from *K. obovata*, which averages less than one copy. Also, t1pks gene cluster are more abundant in mangrove fungi, and the indole gene clusters were significantly different between the fungi isolated from *K. obovata* and *A. ilicifolius*. We speculate that the challenging environment of the mangrove ecosystem has an influence on the diversity of the fungal species, as well as the diversity in the carbohydrate active enzymes and secondary metabolite gene clusters.

## Data Availability Statement

The sequenced data and genome resources in this study were deposited in the CNSA database (https://db.cngb.org/cnsa/) of CNGBdb with accession number CNP0000910.

## Author Contributions

XL, WZ, and GF directed the study. JC, QG, and CS performed data analysis. JW, LP, and JS investigated the materials. WG and QX performed experiments. CS wrote the manuscript. XL and JC performed review and editing. All authors contributed to the article and approved the submitted version.

## Conflict of Interest

All authors are employed by BGI.

## Footnotes

^1^A. F. A. Smit, R.H.P.G. RepeatMasker at http://repeatmasker.org.

^2^A. F. A. Smit. R.H.P.G. RepeatModeler Open-1.0. at http://www.repeatmasker.org/RepeatModeler/.
