# Supplementary material for: Draft Genomes and Comparative Analysis of Seven Mangrove Rhizosphere-Associated Fungi Isolated From Kandelia obovata and Acanthus ilicifolius
Source: Front Fungal Biol. 2021 Apr 14;2:626904. doi: 10.3389/ffunb.2021.626904 (PMC10512393; doi:10.3389/ffunb.2021.626904)
Supplement: Supplementary file 1 [file Data_Sheet_1.ZIP › Supplemental Materials/Supplementary Figures.docx]

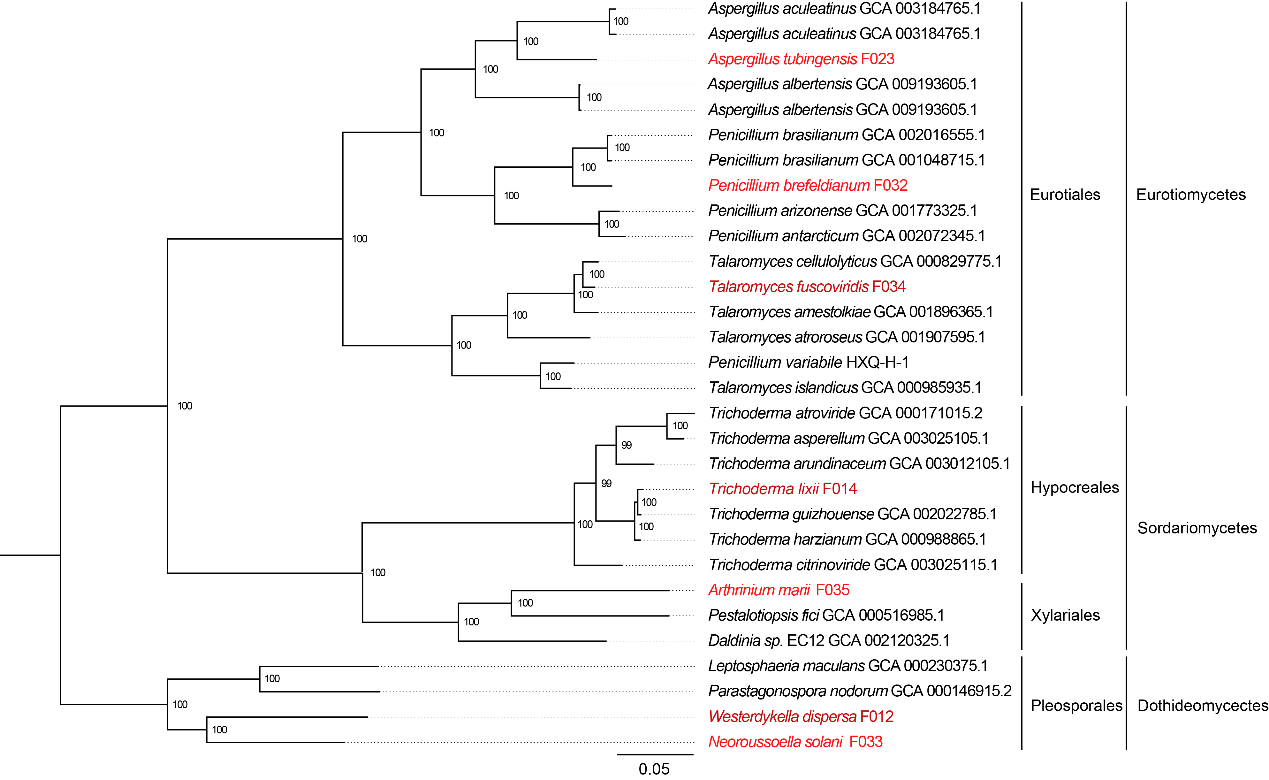


Supplementary Figure 1. Phylogenetic tree based on core genes.


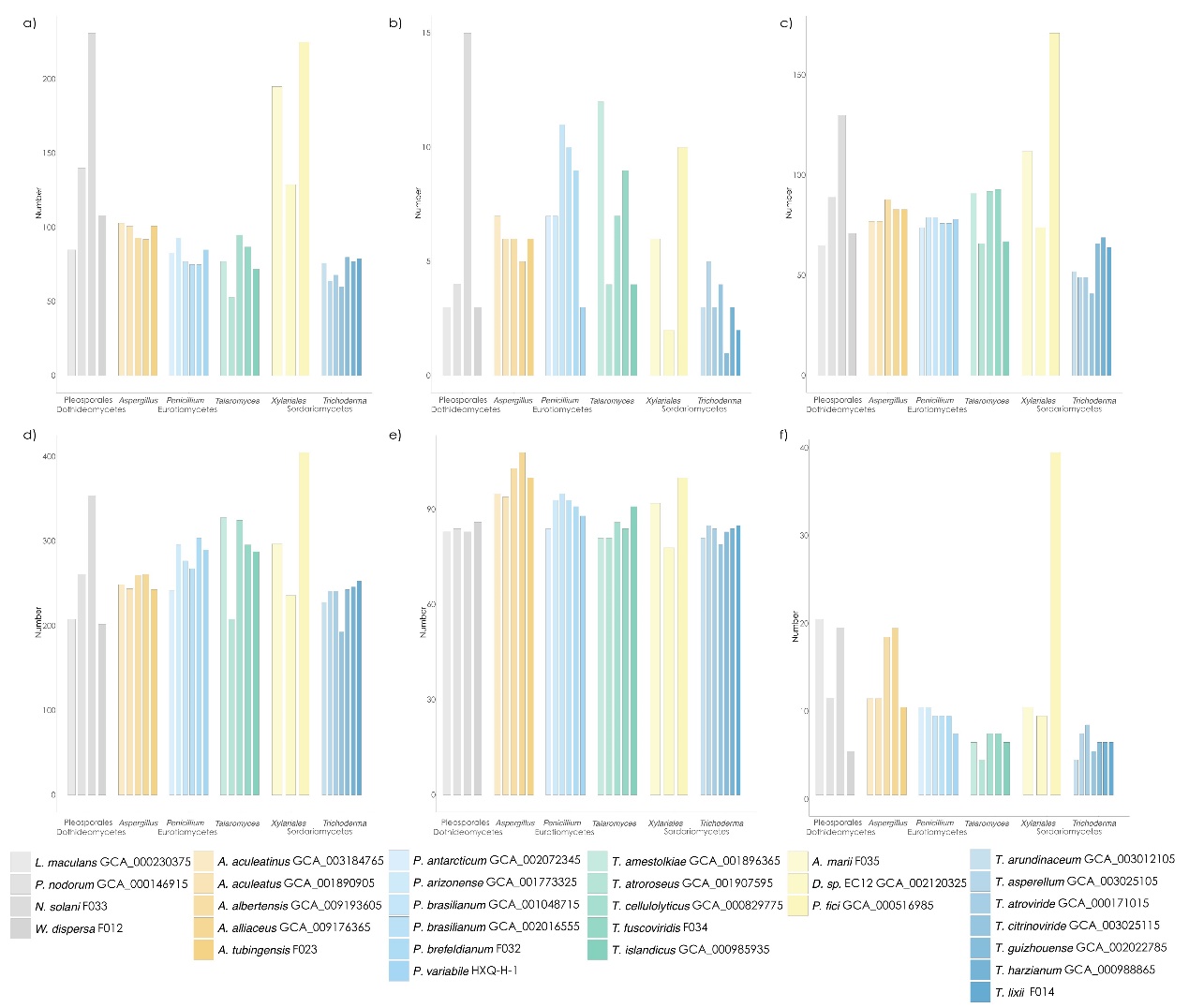


Supplementary Figure 2. Total active enzyme gene numbers of (a) AAs (b) CBMs (c) CEs (d) GHs (e) GTs (f) PLs in each fungus.

Supplementary Figure 3. Comparison of secondary metabolite biosynthesis gene numbers in the mangrove and non-mangrove fungi.

Supplementary Figure 4. Comparison of secondary metabolite biosynthesis gene numbers in the *Kandelia obovate*-derived and *Acanthus ilicifolius*-derived fungi.
